# Improved cell surface display of *Salmonella enterica* serovar Enteritidis antigens in *Escherichia coli*

**DOI:** 10.1186/s12934-015-0227-3

**Published:** 2015-04-09

**Authors:** Martin Gustavsson, Thi-Huyen Do, Petra Lüthje, Ngoc Tan Tran, Annelie Brauner, Patrik Samuelson, Nam Hai Truong, Gen Larsson

**Affiliations:** Royal Institute of Technology (KTH), Division of Industrial Biotechnology, AlbaNova University Center, SE 10691 Stockholm, Sweden; Institute of Biotechnology, Vietnam Academy of Science and Technology, 18-Hoang Quoc Viet, Cau Giay, Ha Noi, Vietnam; Department of Microbiology, Tumor and Cell Biology, Division of Clinical Microbiology, Karolinska Institute and Karolinska University Hospital, Stockholm, Sweden

**Keywords:** Autotransport, Surface expression, *Escherichia coli*, AIDA-I, Live vaccines, *Salmonella enterica*

## Abstract

**Background:**

*Salmonella enterica* serovar Enteritidis (SE) is one of the most potent pathogenic *Salmonella* serotypes causing food-borne diseases in humans. We have previously reported the use of the β-autotransporter AIDA-I to express the *Salmonella* flagellar protein H:gm and the SE serotype-specific fimbrial protein SefA at the surface of *E. coli* as live bacterial vaccine vehicles. While SefA was successfully displayed at the cell surface, virtually no full-length H:gm was exposed to the medium due to extensive proteolytic cleavage of the N-terminal region. In the present study, we addressed this issue by expressing a truncated H:gm variant (H:gmd) covering only the serotype-specific central region. This protein was also expressed in fusion to SefA (H:gmdSefA) to understand if the excellent translocation properties of SefA could be used to enhance the secretion and immunogenicity.

**Results:**

H:gmd and H:gmdSefA were both successfully translocated to the *E. coli* outer membrane as full-length proteins using the AIDA-I system. Whole-cell flow cytometric analysis confirmed that both antigens were displayed and accessible from the extracellular environment. In contrast to H:gm, the H:gmd protein was not only expressed as full-length protein, but it also seemed to promote the display of the protein fusion H:gmdSefA. Moreover, the epitopes appeared to be recognized by HT-29 intestinal cells, as measured by induction of the pro-inflammatory interleukin 8.

**Conclusions:**

We believe this study to be an important step towards a live bacterial vaccine against *Salmonella* due to the central role of the flagellar antigen H:gm and SefA in *Salmonella* infections and the corresponding immune responses against *Salmonella.*

**Electronic supplementary material:**

The online version of this article (doi:10.1186/s12934-015-0227-3) contains supplementary material, which is available to authorized users.

## Background

Bacterial surface display of recombinant proteins has been used for various applications in microbiology, molecular biology, biotechnology and vaccinology [1,2]. The field is dominated by display in *Escherichia coli*, mainly due to the extensive knowledge concerning the genetics, the presence of transformation protocols, the rapid cell growth on simple media, and the multitude of production techniques and protocols for recombinant protein production that are associated with this organism. Further, a range of specific mutant strains with desirable features is available for metabolic engineering.

An area of particular interest is the display of immunogenic peptides at the cell surface for direct use as live vaccine delivery vehicles [2]. This technique offers some interesting advantages over conventional vaccines the first being that a surface display system provides the prerequisites for a more economic process [3]. The reason is that it is designed to circumvent the step of antigen purification and relies on a one-step combination of the production and purification processes. Secondly, the live vaccine systems may have in-built adjuvant effects from endogenous components of the host cell e.g. from the distinctive structure of the *E. coli* lipopolysaccharides (LPS) but also the lipoteichoic acids, peptidoglycans, lipoproteins etc., which are well recognized by the host’s immune system [4] and therefore elicit strong immune responses. Finally, these vehicles may have prolonged retention times due to colonization effects and they are probably also safer to use than inactivated or attenuated pathogen-based vaccines, which may revert to their pathogenic form.

**Figure 1:**
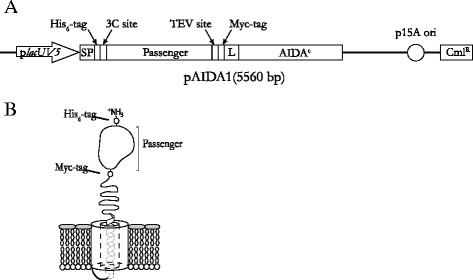
**Schematic representation of the recombinant autodisplay system pAIDA1. A)** The surface display plasmid pAIDA1 based on the AIDA-I autotransporter. Expression is under control of the lacUV5 promoter, and two detection/affinity tags (His_6_ and c-Myc), in addition to two different protease cleavage recognition sites (3C and TEV), flank the passenger protein, which in this study is represented by various *Salmonella* antigens. SP represents the signal peptide, which is cleaved off upon translocation over the inner membrane, while L is the endogenous linker region of AIDA-I (consisting of the first 54 amino acids of the native passenger). The plasmid carries a p15A origin of replication and a chloramphenicol resistance gene (Cml^R^). **B)** Outer membrane (OM) including the surface displayed recombinant protein (mature) expressed from pAIDA1.

Surface display relies today on a number of different techniques. We have on several occasions successfully used the autotransporter AIDA-I (Adhesin Involved in Diffuse Adherence of enteropathogenic *E. coli*) [5] to express and display different peptides and proteins on the cell surface of a laboratory *E. coli* K-12 strain into which the AIDA-inherent system of pathogenic *E. coli* was transfered [6-8]. Our autotransporter vector pAIDA1 consists of a specific N-terminal signal peptide followed by a passenger protein, a linker region and a C-terminal translocation unit, AIDA^C^, which forms a β-barrel-type outer membrane (OM) pore [9,10], as illustrated in Figure 1. The signal peptide is cleaved off after the Sec-mediated translocation over the inner membrane, followed by insertion and formation of the β-barrel pore through which the linker and passenger moiety eventually pass to become surface exposed [9,10]. Recently, we expressed the *Salmonella enterica* serovar Enteritidis (SE) proteins SefA and H:gm at the *E. coli* cell surface [7,11,12] to investigate the potential of the surface expression technique as a process for live vaccine production. Although two different versions of the recombinant AIDA-system allowed us to express both the flagellar protein H:gm and the fimbrial protein SefA in *E. coli*, only SefA was successfully exposed as a full-length protein at the surface [7,11]. Western blot analysis showed the presence of H:gm in the OM fraction but revealed that it was proteolytically cleaved.

**Figure 2:**
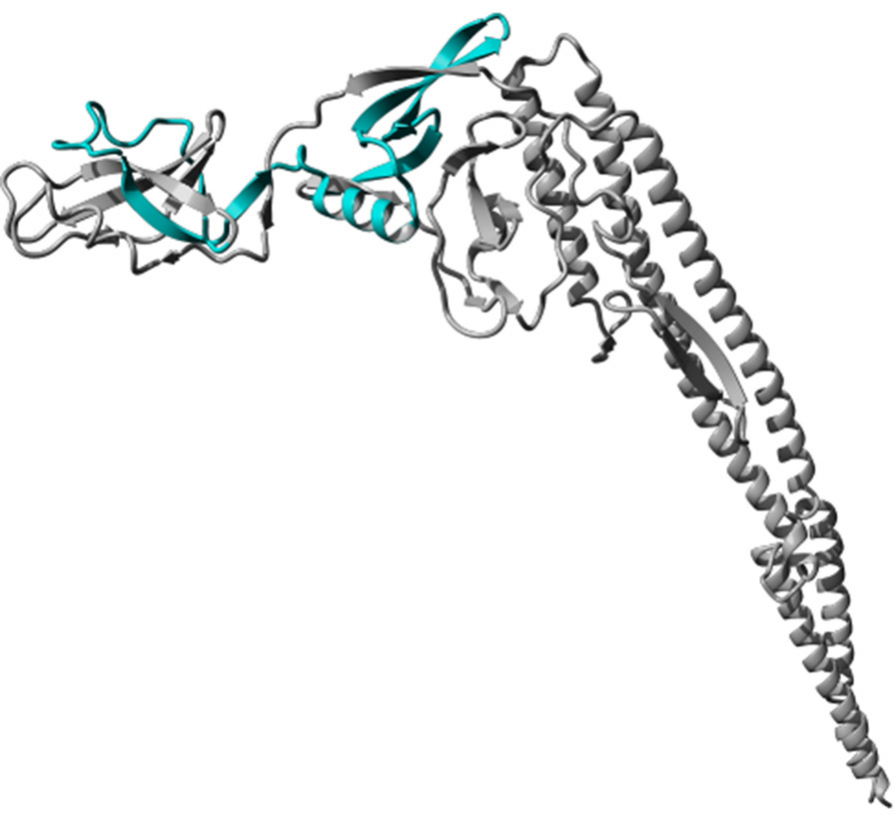
**3D-structure of H:gm.** Atomic model of the *Salmonella enterica* flagellar antigen H:gm (507 aa) generated with Phyre2 (Protein Homology/Analogy Recognition Engine) [26] and drawn in PyMOL. The protein forms a tubular like structure with the N- and C-terminal ends in close proximity to each other. The region corresponding to H:gmd (aa 254–351) is highlighted in cyan.

It is well known that SefA and H:gm both play central roles in *Salmonella* infections and elicit a protective immune response against SE [13-17]. Hence, the inability to express and display H:gm as a full-length protein on the *E. coli* cell surface might reduce the efficacy of this strain as a live vaccine. We hypothesized that our previous difficulties of surface expression of H:gm, and the partial proteolysis observed, was due either to the larger size of this protein compared to SefA or due to premature folding of the quite complex structure of the H:gm protein in the OM both leading to a longer exposure time to periplasmic and OM proteases. In the present investigation we addressed this issue by protein engineering reducing H:gm into a smaller derivative consisting of only the serotype-specific region [18], denoted H:gmd (Figure 2). This smaller unit was expressed by the AIDA-I vector but also in fusion to SefA with the idea to boost the production by use of a protein with known and excellent translocation properties, as well as with the aim to produce a vaccine targeting both epitopes using a single cell.

## Results

### Surface expression of H:gm, H:gmd, SefA, and H:gmdSefA in *E. coli*

*E. coli* O17, with the deletion of the outer membrane protease OmpT, was used for production to avoid the cleavage of the protein from the cell surface that would otherwise take place [6]. This strain was transformed with the surface expression plasmid pAIDA1, which was used to produce the engineered variant of the *Salmonella* flagellar protein H:gm, here denoted H:gmd. In addition, this protein was also fused to the fimbrial protein SefA to investigate a supposed positive effect on production by utilizing the known successful translocation of this particular protein. For comparison, strains producing the full-length protein H:gm, as well as SefA, were cultivated and the expression compared to the new constructs. A comparison of the properties of these xproteins can be found in Table 1. As a zero-reference, *E. coli* growth without the plasmid was used. All processes were performed by batch technology and sampled during the logarithmic phase for subsequent analysis.

**Table 1:** **Description of the different passenger proteins expressed in this study**

**Protein**	**Description**	**Size (kDa)**	**Length (aa)**	**Reference**
SefA	Fimbrial subunit	14.5	144	12
H:gm	Flagellar subunit	53.2	508	12
H:gmd	H:gm epitope	11.0	111	This study
H:gmdSefA	H:gmd-SefA fusion	25.4	254	This study

As seen in Figure 1, the pAIDA1 vector is constructed to harbor two distinct tags, His_6_ and c-Myc that are flanking the recombinant passenger protein on the N- and C-terminal sides, respectively. Using fluorescently labeled antibodies specific for the tags allows us to determine the presence and integrity of the different fusion proteins using flow cytometry.

**Figure 3:**
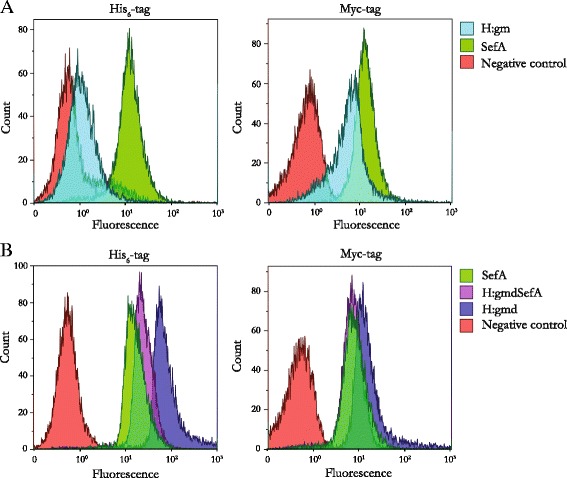
**Flow cytometry histograms of various** ***Salmonella*** **antigens expressed on the surface of** ***E. coli*** **.** The presence of the His_6_-tag and c-Myc-tag in the displayed proteins were probed with THE™ His Tag Antibody [FITC] and Anti-c-Myc [SureLight® Allophycocyanin] antibody, respectively. Negative controls (cells lacking surface display plasmids) are all shown in red. **(A)** Left panel: His-probed *E. coli*/pAIDA1-H:gm (light blue) and *E. coli*/pAIDA1-SefA (green). Right panel: c-Myc-probed *E. coli*/pAIDA1-H:gm (light blue) and *E. coli*/pAIDA1-SefA (green). **(B)** Left panel: His-probed *E. coli*/pAIDA1-H:gmd (dark blue), *E. coli*/pAIDA1-SefA (green), and *E. coli*/pAIDA1-H:gmdSefA (purple). Right panel: c-Myc-probed *E. coli*/pAIDA1-H:gmd (dark blue), *E. coli*/pAIDA1-SefA (green), and *E. coli*/pAIDA1-H:gmdSefA (purple).

All recombinant fusion proteins showed whole-cell fluorescence intensities greater than the negative control when probed with the anti-c-Myc-antibody (Figure 3), which is located closest to the cell surface, i.e. on the inside with respect to the surface exposed antigen (Figure 1). This confirms that all constructs were expressed at least as far as to the translocation unit (AIDA^C^) with the linker and c-Myc-tag that were inserted and accessible in the outer cell membrane to the binding of the fluorophore.

Figure 3 shows also the data for the second detection system based on the His_6_-tag that is situated outside the antigen facing the medium. All constructs, except H:gm (Additional file 1: Figure S1), stained positive for the presence of this tag at the cell surface. The fluorescence intensity of the H:gm-expressing cells was comparable to the negative control while the new constructs exhibited a significant increase in fluorescence. In contrast to H:gm, the novel construct containing the smaller flagellar antigen H:gmd was expressed very well at the cell surface. In fact, this construct resulted in the highest fluorescence of all constructs tested for both the anti-His_6_ and the anti-c-Myc antibody (Figure 3B).

### Subcellular localization and size of the fusion proteins

**Figure 4:**
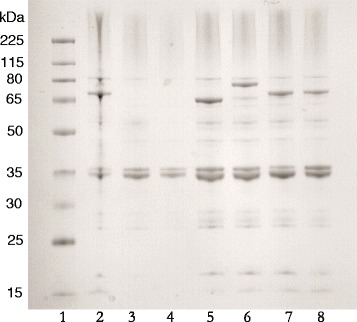
**SDS-PAGE analysis of recombinant** ***Salmonella*** **antigens recovered from** ***E. coli*** **outer membrane fractions.** Lane 1: Size marker (Spectra Multicolour Broadrange, Fermentas); Lane 2: O17ΔOmpT/pAIDA1-SefA (positive control; shake flask cultivation from a prior experiment [7]; Lane 3: empty O17ΔOmpT (negative control); Lane 4: non-induced O17ΔOmpT/pAIDA1-SefA (negative control); Lane 5: O17ΔOmpT/pAIDA1-H:gmd; Lane 6: O17ΔOmpT/pAIDA1-H:gmdSefA; Lane 7 & 8: O17ΔOmpT/pAIDA1-SefA, replicates.

To specify the localization and integrity of the different fusion proteins whole-cell lysates were fractioned and the OM fraction was collected for comparison of the different constructs. This fraction was analyzed by SDS-PAGE and the result is shown in Figure 4. As can be seen, the *Salmonella* antigens were correctly expressed in the OM and appeared to generate full-length fusion proteins of the expected sizes; 68.8 kDa (H:gmd-AIDA^C^), 83.3 kDa (H:gmdSefA-AIDA^C^), 72.6 kDa (SefA-AIDA^C^). As anticipated, the OM fractions of the negative controls (wild type *E. coli* O17ΔOmpT or non-induced O17ΔOmpT containing pAIDA1-SefA) did not generate any detectable protein bands corresponding to those of the relevant *Salmonella* antigen fusions. These results thus corroborate well with the whole-cell fluorescence data of Figure 3. Additional bands for the outer membrane proteins OmpF (39 kDa) and OmpA (35 kDa) [19] were observed in all analysed samples.

A maybe even more important finding was that H:gmd did not show any visible signs of proteolytic degradation, neither in the FACS fluorescence data (Figure 3) or by SDS-PAGE analysis (Figure 4), in contrast to the previously used H:gm that gave rise to little full-length protein due to extensive proteolysis (Additional file 1: Figure S1) [7,11]. This is a significant improvement as H:gmd contains the serotype-specific specific amino acids, and thus might increase its potential as a live bacterial subunit vaccine against *Salmonella.*

### Functionality of the expressed proteins

**Figure 5:**
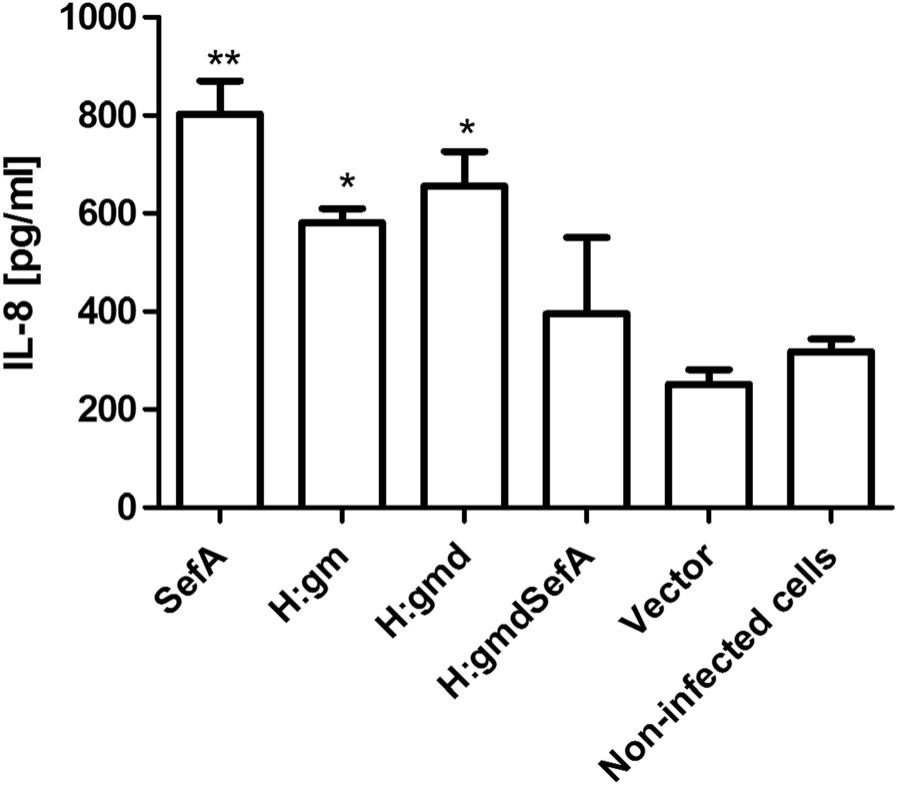
***Salmonella*** **antigens expressed by** ***E. coli*** **induce a pro-inflammatory response in colon epithelial cells.** HT-29 cells were infected with *E. coli* strains expressing SefA, H:gm, H:gmd or H:gmdSefA from *Salmonella,* and IL-8 was measured in the supernatant after 4 hours. *E. coli* with the pAIDA1 plasmid only (vector) and non-infected HT-29 cells served as controls. Results are shown as mean and SEM; comparison with the vector control was performed by ANOVA and Dunnett’s multiple comparison test, *P < 0.05, **P < 0.01.

Having verified their correct localization and surface exposure, we finally wanted to check if the engineered antigen H:gmd might still provoke an immunogenic response since a severe deletion was imposed on the full length H:gm protein. It has previously been reported that *S. enterica* flagellar and fimbrial proteins (i.e. SefA and H:gm), can induce an IL-8 response from gut epithelial cell line HT-29 [20]. Thus, an *in vitro* assay was devised by exposing this cell line to *E. coli* expressing the various epitope constructs, while measuring the release of IL-8 as marker for a pro-inflammatory response. An elevated response was detected for the strains expressing SefA, H:gm and H:gmd as compared to the strain carrying a surface expression vector without any of the SE epitopes (Figure 5). The highest response was seen from SefA followed by H:gmd and H:gm, where the two latter were of approximately the same magnitude. Interestingly, there was no significant response to the H:gmdSefA construct. Furthermore, it appears that the chosen *E. coli* host strain was unable to raise an IL-8 response by itself, as the control bearing the empty surface expression vector showed similar or lower IL-8 levels as the uninfected negative control.

## Discussion

The main goal with the present study was to successfully express and display H:gm-derived antigens at the surface of *E. coli* by using the AIDA-I autotransporter system. Our previous attempts to express and display H:gm were unsuccessful due to extensive proteolytic cleavage in the C-terminal region of that antigen [7,11]. To make H:gm less affected to proteolytic degradation could be achieved through several approaches. One option would be the usage of protease-deficient *E. coli* strains. However, we already used a mutant strain deficient for the protease OmpT (O17ΔOmpT) and a clear candidate protease could not be identified from our previous experiments. Instead, we chose to engineer the protein to get a H:gm-derivative that could potentially be efficiently displayed at the cell surface and be less sensitive to proteolytic cleavage. For this purpose, we here chose to include only a subpart, called H:gmd, comprising 98 aa out of the total 507 aa in the full-length H:gm (Figure 2). The rationale behind this was threefold. Firstly, the chosen region comprises the serotype-specific part of H:gm needed for development of immunity to SE [21,22]. Secondly, the large size of H:gm can be expected to lead to a longer time required for full translocation over the outer membrane, resulting in an extended exposure to periplasmic proteases. By expressing only a small part of H:gm this exposure should be reduced and consequently the protein should be less susceptible to proteolysis. Thirdly, it has been shown that passenger proteins that form stable folds in the periplasm, for instance due to disulphide formation, are difficult to display using autotransporters due to stalled translocation over the outer membrane [23,24]. As seen in Figure 2, H:gm folds with the N-and C-terminals in close proximity, which could be problematic given the autotransporter translocation mechanism. By expressing only the H:gmd domain this potentially problematic part of the protein is excluded.

This strategy proved to be very successful, as H:gmd generated whole-cell fluorescence intensities much greater than the negative control, both when the presence of the N-terminal His_6_-tag and the C-terminal c-Myc tag were analyzed (Figure 3B). This is in contrast to the original H:gm construct, which did not generate a signal above the negative control when probed with the His_6_-reactive antibody, indicating proteolytic degradation (Figure 3A, left panel). Interestingly, not only was the smaller H:gmd protein expressed to full-length at the cell surface, but it also appeared to promote the display and accessibility of the fused protein H:gmdSefA since H:gmd and H:gmdSefA both resulted in higher fluorescence intensities than SefA alone or the full-length H:gm. Although the true reasons behind the observed improvements in expression and cell surface display characteristics of H:gmd over H:gm at present are unclear, they can likely be attributed either to structural reasons as discussed previously, or that the protease sensitive amino acids in H:gm are not present in H:gmd.

With the ultimate goal to generate suitable tools for live vaccine development, we tested the *E. coli* strains expressing the different *Salmonella* epitopes for their ability to induce an immune reaction in gut epithelial cells. The response in the *in vitro* system applied here was overall very low, probably related to the low immunogenicity of the non-pathogenic *E. coli* K-12 strain O17ΔOmpT, which was unable to stimulate IL-8 production on its own. However, our results suggest that while SefA appeared the most potent immunogen, the truncated H:gmd variant was to some extent even superior to the full-length H:gm construct, in line with the flow cytometric resultss. In contrast, the fusion construct H:gmdSefA did not perform well in this assay. A possible explanation might be that the epitopes of the single proteins are hidden by the structure of the fusion protein.

## Conclusions

In this study we engineered a truncated variant of H:gm comprising only the serotype-specific amino acids. H:gmd was resistant to proteolytic cleavage and exhibited highly efficient surface display, in contrast to H:gm. This is a promising result from the perspective of creating a live vaccine against SE, though additional studies are needed to further confirm the immunogenicity of these constructs and to investigate their ability to raise SE-specific antibodies in an animal host.

## Methods

### Bacterial strains and plasmids

*Escherichia coli* K-12 strain O17ΔOmpT was used for surface expression using the AIDA-I autotransporter, as previously reported [6]. The plasmid pAIDA1-SefA [7] was used to produce a recombinant fusion of SE fimbriae protein SefA and AIDA-I on the surface of the host cell. This plasmid encodes the C-terminal AIDA β-barrel (AIDA^C^) followed by a linker consisting of the first 54 amino acids (aa) of the native AIDA passenger, the recombinant SefA passenger and finally the native AIDA signal peptide. Additionally, the plasmid contains two epitope tags (c-Myc and His_6_) located on the C- and N-terminal sides of SefA, respectively (Figure 1). pAIDA1-H:gm [6-8] encodes the SE flagellar antigen H:gm instead of SefA. For construction of pAIDA1-H:gmd and pAIDA1-H:gmdSefA, standard recombinant DNA techniques were used [25]. Restriction endonucleases, T4 DNA ligase, plasmid purification kits (miniprep), and DreamTaq DNA polymerase were purchased from Fermentas. Primers were supplied by Eurofins MWG Operon while GE Healthcare supplied the PCR DNA and Gel band Purification Kit. H:gmd_forw (5′-acaggtaccactaaatctactgctggaaccgctgaag-3′) and H:gmd_rev (5′-actgagctcctggatccacgaccctccgcctcctgaacccccgcctccgtttttggttttatcatcaaaag-3′) were used to PCR amplify a H:gmd-encoding DNA fragment from pAIDA1-H:gm. The resulting amplicon was then purified, digested with KpnI and SacI, gel purified and finally ligated into KpnI- and SacI-digested pAIDA1 to generate pAIDA1-H:gmd. To create pAIDA1-H:gmdSefA, the purified H:gmd-amplicon was instead cleaved with KpnI and BamHI, gel purified and then ligated into pAIDA1-SefA previously digested with KpnI and BclI, thus yielding pAIDA1-H:gmdSefA. These novel constructs were all verified by DNA sequencing. Table 1 shows a summary of the properties of the different antigens.

### Cultivation

All cultivations were performed in minimal salts medium, consisting of (per liter): 7.0 g (NH_4_)_2_SO_4_, 1.6 g KH_2_PO_4_, 6.6 g Na_2_HPO_4_^.^2H_2_O, and 0.5 g (NH_4_)_2_-H-Citrate. In addition, all media were supplemented with 1 ml l^−1^ of both 1 M MgSO_4_ and trace element solution that were sterile filtered into the different reactors. The trace element stock solution consisted of (per liter): 0.5 g CaCl_2_ · 2H_2_O, 16.7 g FeCl_3_ · 6H_2_O, 0.18 g ZnSO_4_ · 7H_2_O, 0.16 g CuSO_4_ · 5H_2_O, 0.15 g MnSO_4_ · 4H_2_O, 0.18 g CoCl_2_ · 6H_2_O, 20.1 g Na-EDTA.

All cultivations were performed in a batch format initialized by shake flask growth in 1 L bottles containing 100 ml of minimal salts medium supplemented with 10 g l^−1^ glucose. These were inoculated from a frozen glycerol stock (−80°C) and placed inside a shaking incubator (37°C, 180 rpm) over night. In the following morning, six samples were withdrawn and used to inoculate a six-parallel stirred-tank bioreactor unit (Greta, Belach Bioteknik AB, Sweden) where each reactor contained 800 ml of minimal medium to which 10 g l^−1^ of separately sterilized glucose had been added. After inoculation, the cultures were grown for two generations to an optical density of 0.4, before induction by addition of isopropyl β-D-1-thiogalactopyranoside (IPTG) to a concentration of 200 μM. Zero-samples for surface expression were withdrawn immediately before induction. The cultures were then grown for approximately four generations, after which final samples were withdrawn for subsequent analysis and comparison.

The dissolved oxygen concentration (DOT) in the reactors was controlled at 40% by automatic adjustment of the stirrer speed. The airflow was initially set to 0.1 VVM, and then incrementally increased to 1.0 VVM. The temperature was automatically controlled at 37°C and pH was kept at 7.0 by titration with NH_4_OH (12.5% w/v). Foaming was kept at a minimum by manual addition of antifoaming agent when necessary.

### Analyses

#### Cell mass

Cell mass accumulation was followed by sampling at regular intervals. The optical density at 600 nm (OD_600_) was measured using a spectrophotometer (Genesys 20, Thermo Scientific). All samples were diluted in saline solution (0.9% NaCl) to an approximate OD_600_ of 0.1 prior to measurement to compensate for the non-linearity of OD_600_ measurements, and actual OD_600_ values were derived by multiplication with the dilution factor.

#### Flow cytometric analyses of surface expression

Cell samples for analysis of surface expression were aseptically withdrawn and stored at −80°C, as previously described [6]. At the day of analysis, 50 μl of each sample was labeled using fluorescent antibodies against the two detection tags (His_6_ and Myc) flanking the passenger protein, as described previously [7]. Surface expression levels were evaluated based on measurement of fluorescence from the two antibodies using a flow cytometer (Gallios, Beckman Coulter). 10,000 events per sample were recorded. The excitation wavelength was 488 nm for the His_6_-tag (FITC) analysis and emission was detected at 525/40 nm, while the c-Myc-analysis (SureLight® Allophycocyanin) used 638 nm and 660/20 nm for the excitation and emission wavelengths, respectively.

#### Analysis of OM proteins

*E. coli* proteins were separated into soluble, inner membrane and OM fractions as previously reported [19]. The resulting outer membrane protein samples were separated using SDS-PAGE on 10 % Bis-Tris gels (NuPage, Invitrogen). The gels were then stained using PageBlue protein staining solution (Fermentas) according to the manufacturer’s protocol.

#### Cell stimulation assay

Colonrectal adenocarcinoma cell line HT-29 (ATCC, HTB-38) were grown in McCoy’s 5A medium supplemented with 10% fetal bovine serum at 37°C in a humidified incubator with 5% CO_2_. For experiments, cells were seeded on 24-well plates and used when confluent. Bacteria were collected at the time point of optimal protein expression by centrifugation, washed once in phosphate-buffered saline (PBS) and adjusted to an OD_600_ of 0.125 in PBS. Bacteria were diluted 1:100 in complete cell culture medium to reach a final concentration of approximately 10^6^ colony forming units (CFU)/ml. Medium was removed from the cells and 1 ml of fresh medium with bacteria was added. The plate was centrifuged at 600 *g* for 5 min to accelerate bacterial contact to the cells and correct for potential differences in motility of the strains. The infected cells were incubated at 37°C in a humidified incubator with 5% CO_2_ for 4 hours. Then, the supernatants were collected, floating cells were removed by centrifugation at 300 *g* for 5 min and the cleared supernatants were stored at −80°C until analysis. Secreted interleukin (IL) 8 was quantified by enzyme-linked immunosorbent assay (ELISA) according to the manufacturer’s protocol (R&D Systems).

## Additional file

Additional file 1: Figure S1. Flow cytometric analysis of *E. coli* O17ΔOmpT expressing pAIDA1-H:gm (green) and pAIDA1-SefA (blue). Significant N-terminal proteolysis of H:gm was evident from the low signal from the N-terminal His6-tag (A) relative to the C-terminal Myc-tag (B). Red: O17ΔOmpT without the surface display vectors (negative control). Reproduced from Jarmander et al. 2012 [7].
