# Supplementary material for: Improved cell surface display of Salmonella enterica serovar Enteritidis antigens in Escherichia coli
Source: Microb Cell Fact. 2015 Apr 9;14:47. doi: 10.1186/s12934-015-0227-3 (PMC4415288; doi:10.1186/s12934-015-0227-3)
Supplement: Additional file 1: Figure S1. — Flow cytometric analysis of E. coli O17ΔOmpT expressing pAIDA1-H:gm (green) and pAIDA1-SefA (blue). Significant N-terminal proteolysis of H:gm was evident from the low signal from the N-terminal His6-tag (A) relative to the C-terminal Myc-tag (B). Red: O17ΔOmpT without the surface display vectors (negative control). Reproduced from Jarmander et al. 2012 [7]. [file 12934_2015_227_MOESM1_ESM.docx]

**Figure S1** Flow cytometric analysis of *E. coli* 0:17ΔOmpT expressing pAIDA1-H:gm (green) and pAIDA1-SefA (blue). Significant N-terminal proteolysis of H:gm was evident from the low signal from the N-terminal His6-tag (A) relative to the C-terminal Myc-tag (B). Red: 0:17ΔOmpT without the surface display vectors (negative control). Reproduced from Jarmander et al. 2012[1].

1. Jarmander J, Gustavsson M, Do T-H, Samuelson P, Larsson G: **A dual tag system for facilitated detection of surface expressed proteins in *Escherichia coli*.** *Microb Cell Fact* 2012, **11**:118.
